# Supplementary material for: Global transcriptomics and targeted metabolite analysis reveal the involvement of the AcrAB efflux pump in physiological functions by exporting signaling molecules in Photorhabdus laumondii
Source: Microbiol Spectr. 2025 Sep 8;13(10):e01106-25. doi: 10.1128/spectrum.01106-25 (PMC12502680; doi:10.1128/spectrum.01106-25)
Supplement: Supplemental material — Fig. S1 to S6; Tables S1 to S4. [file spectrum.01106-25-s0001.pdf]

Supplementary Materials

Global Transcriptomics and Targeted Metabolite Analysis Reveal the Involvement of the AcrAB Efflux Pump in Physiological Functions by Exporting Signaling Molecules in *Photorhabdus laumondii* TT01.

Linda Hadchity, Anne Lanois-Nouri, Adrien Chouchou, David Roche, Jessica Houard, Noémie Claveyroles, Alexandra Dauvé, Jacques Imbert, Maxime Gualtieri, Alain Givaudan*, Alyssa Carré-Mlouka*, and Ziad Abi Khattar*.

This PDF file includes:

Supplementary Figures 1 to 6

Supplementary Tables 1 to 4

Supplementary References

Supplementary Figures

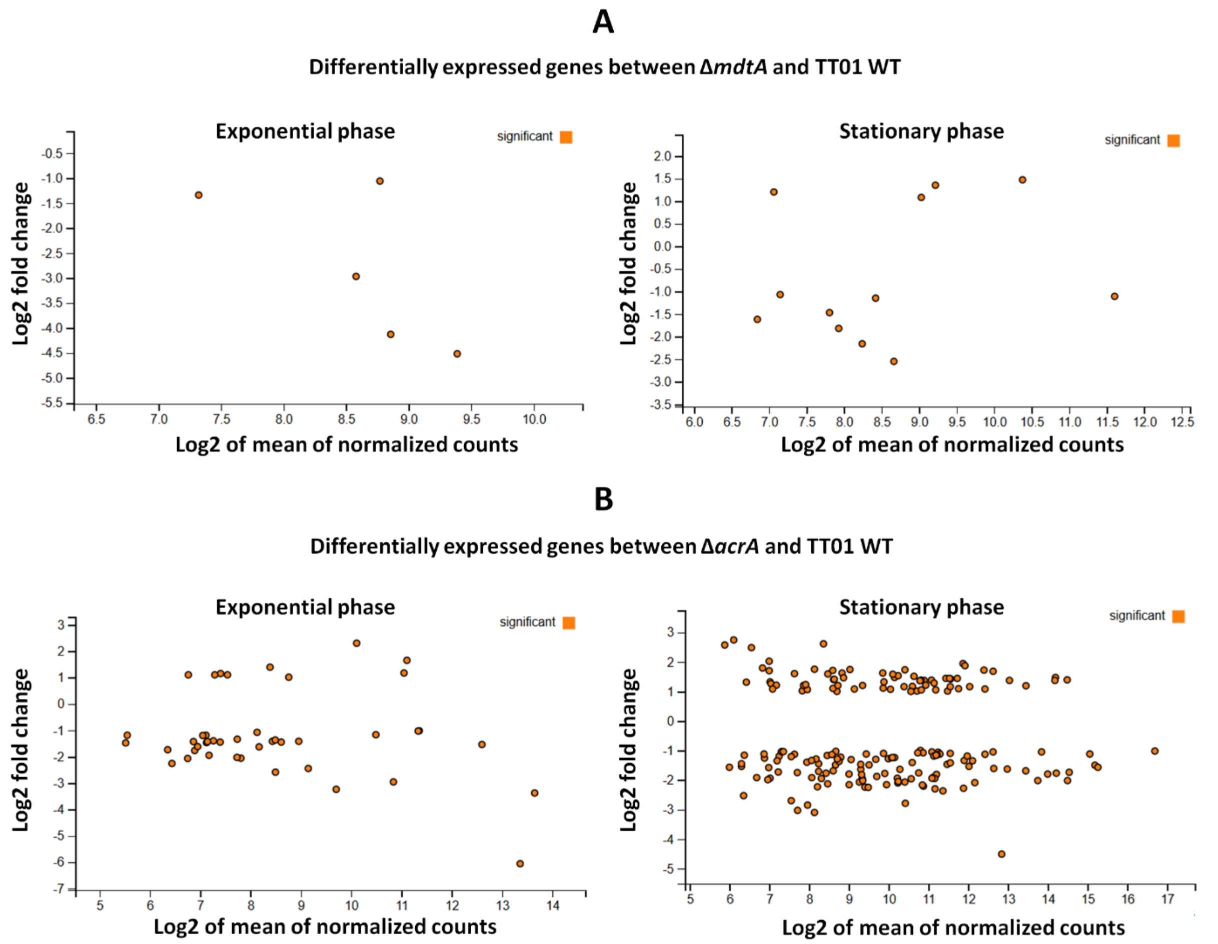

Fig. S1. Overview of genes significantly differentially expressed by RNA sequencing (RNA-seq) analysis of *P. laumondii* strains. RNA-seq analysis between the WT with 5086 total number of genomic objects (GO) and (A) Δ*mdtA,* and (B) Δ*acrA* cultured to exponential (OD_{540} = 0.5-0.7) and stationary (OD_{540} = 2.3-4.2) growth phases in LB broth medium.

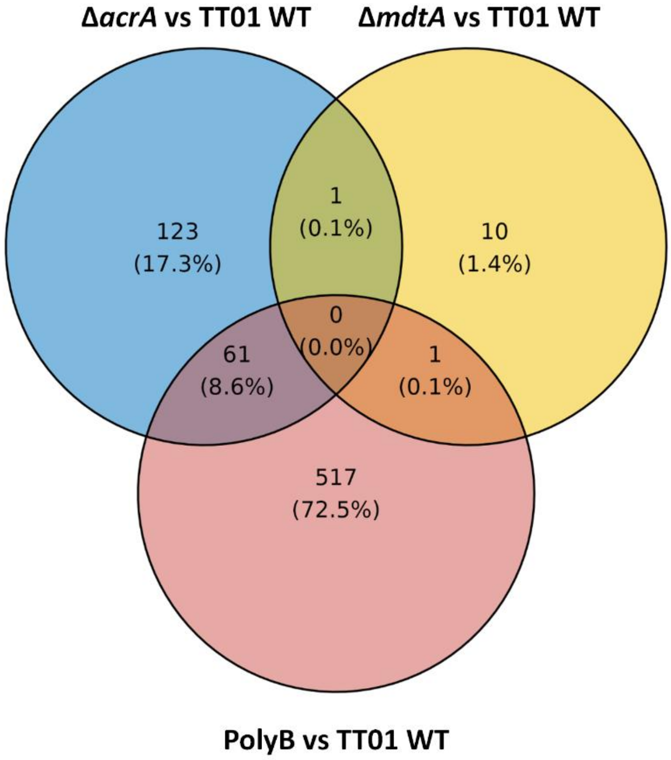

Fig. S2. Venn diagram of significantly differentially expressed genes between *P. laumondii* strains. The Venn diagram was performed using R package ggvenn v0.1.16 (1) and R environment v4.1.1 (2) and compares transcriptomic data from Δ*acrA* and Δ*mdtA* mutants versus WT grown in LB to stationary growth phase (OD_{540} = 2.3-4.2), and WT grown in LB with polymyxin B versus WT grown in LB (3). Percentages correspond to the number of genes differentially expressed between WT and mutant strains, or polymyxin B-resistant subpopulation out of a total of 713 genes corresponding to the total number of differentially expressed genes of the three RNA-seq analyses (Δ*mdtA*, Δ*acrA*, and a polymyxin B-resistant subpopulation of *P. laumondii* TT01) versus WT.

58

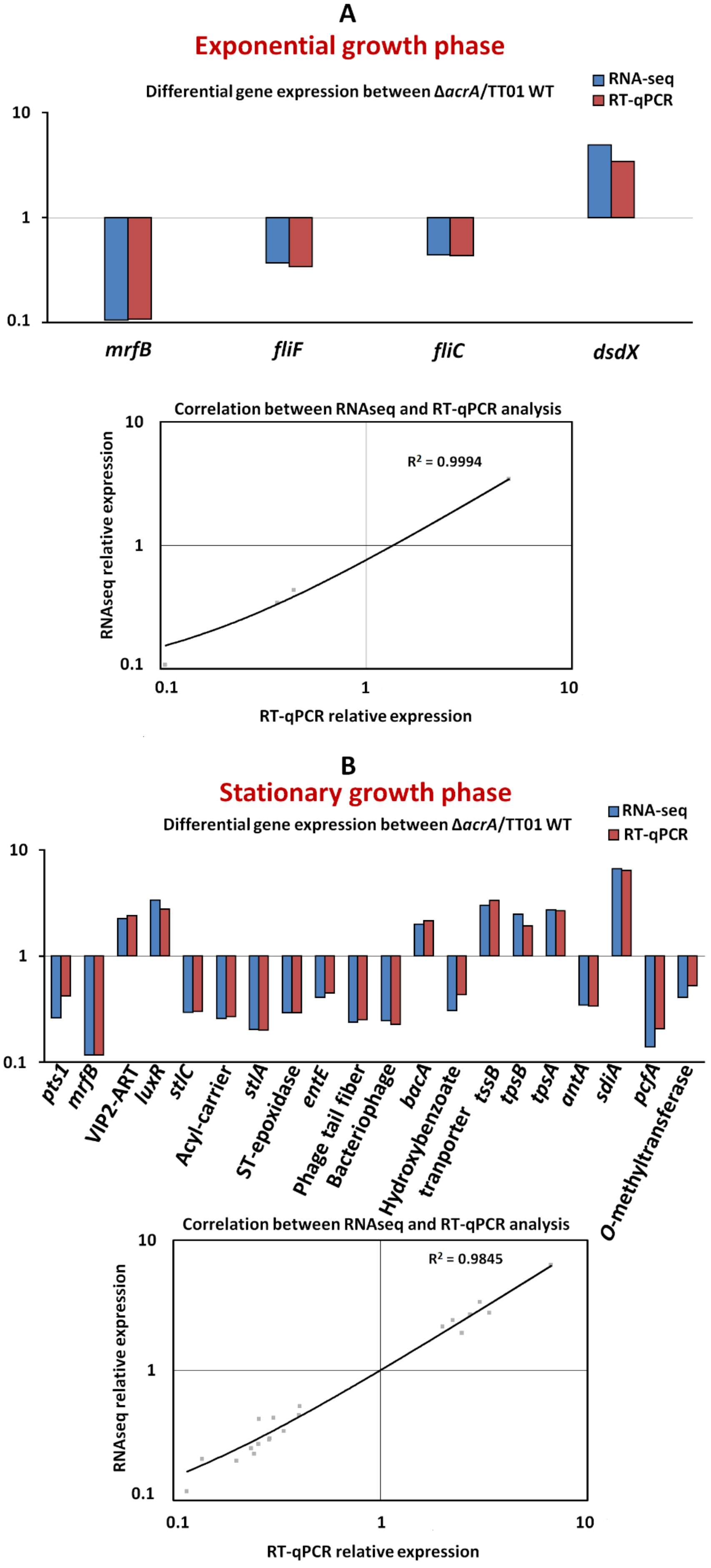
A
Exponential growth phase
Differential gene expression between ΔacrA/TT01 WT
RNA-seq
RT-qPCR
10
1
0.1
mrfB
fliF
fliC
dsdX
Correlation between RNAseq and RT-qPCR analysis
R² = 0.9994
RNAseq relative expression
RT-qPCR relative expression
B
Stationary growth phase
Differential gene expression between ΔacrA/TT01 WT
RNA-seq
RT-qPCR
pts1
mrfB
VIP2-ART
luxR
stlC
Acyl-carrier
stlA
ST-epoxidase
entE
Phage tail fiber
Bacteriophage
bacA
Hydroxybenzoate tranporter
tssB
tpsB
tpsA
antA
sdiA
pcfA
O-methyltransferase
Correlation between RNAseq and RT-qPCR analysis
R² = 0.9845
RNAseq relative expression
RT-qPCR relative expression

Fig. S3. Transcriptional expression of crucial genes differentially expressed by RNA sequencing analysis between *P. laumondii* TT01 WT and Δ*acrA* strains. Total RNA was extracted from the WT and *acrA* mutant strains cultured in LB broth medium to (A) exponential (OD_{540} = 0.5-0.7) and (B) stationary (OD_{540} = 2.3-4.2) growth phases. Comparison between RNA-seq and RT-qPCR results of the differential gene expression between the WT and Δ*acrA*. Histograms show the expression ratios of target genes between the WT and the Δ*acrA* strain normalized to *recA* used as a housekeeping gene, and relative to the WT. Results shown are the medians of three biological experiments with three technical replicates per experiment.

Correlation factors (R^2) between RNA-seq and RT-qPCR analysis for each growth phase were calculated from the log2 fold change ratio (Δ*acrA*/WT) obtained by RNA-Seq against the log2 fold change ratio (Δ*acrA*/WT) obtained by RT-qPCR of 4 genes in exponential phase and 20 genes in stationary phase.

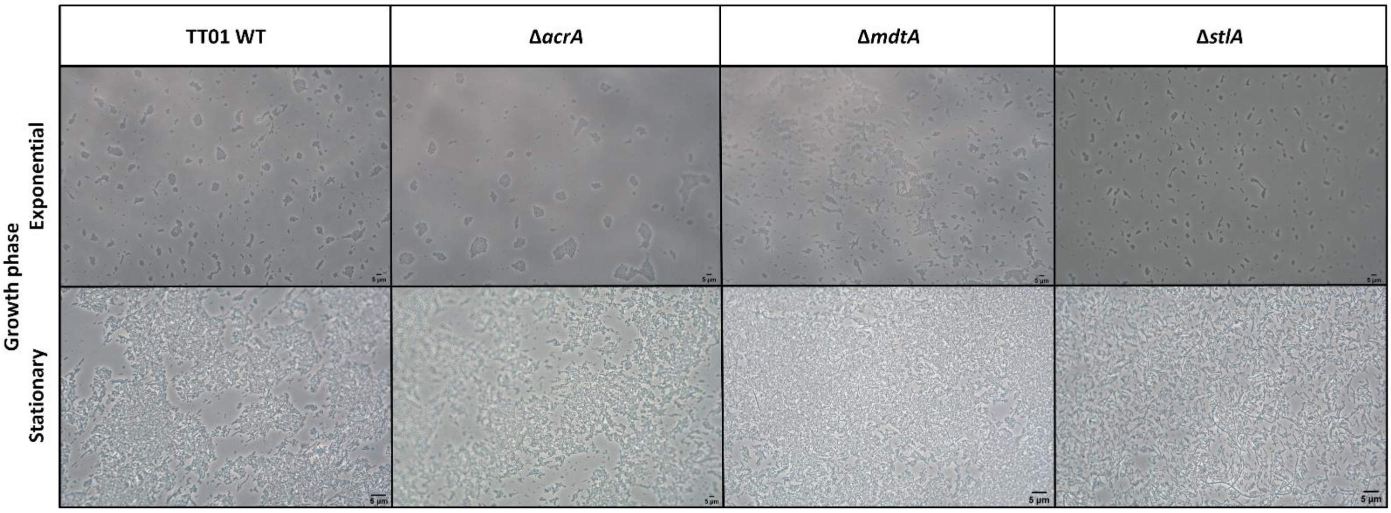

Fig. S4. No clear phenotype was observed for the involvement of AcrAB in cellular clumping in *P. laumondii* TT01. Cellular clumping was assessed using contrast phase- microscopy on three independent biological replicates of *P. laumondii* WT, Δ*acrA*, Δ*mdtA*, and Δ*stlA* strains grown to exponential (OD_{540} = 0.28-0.4) and stationary (OD_{540} = 1.8-4.1) growth phases in LB broth medium at 28°C without shaking. Results shown are representative of one replicate of each strain. Photos show a slight decrease in Δ*stlA* cell clumps compared to WT, Δ*acrA*, and Δ*mdtA* strains.

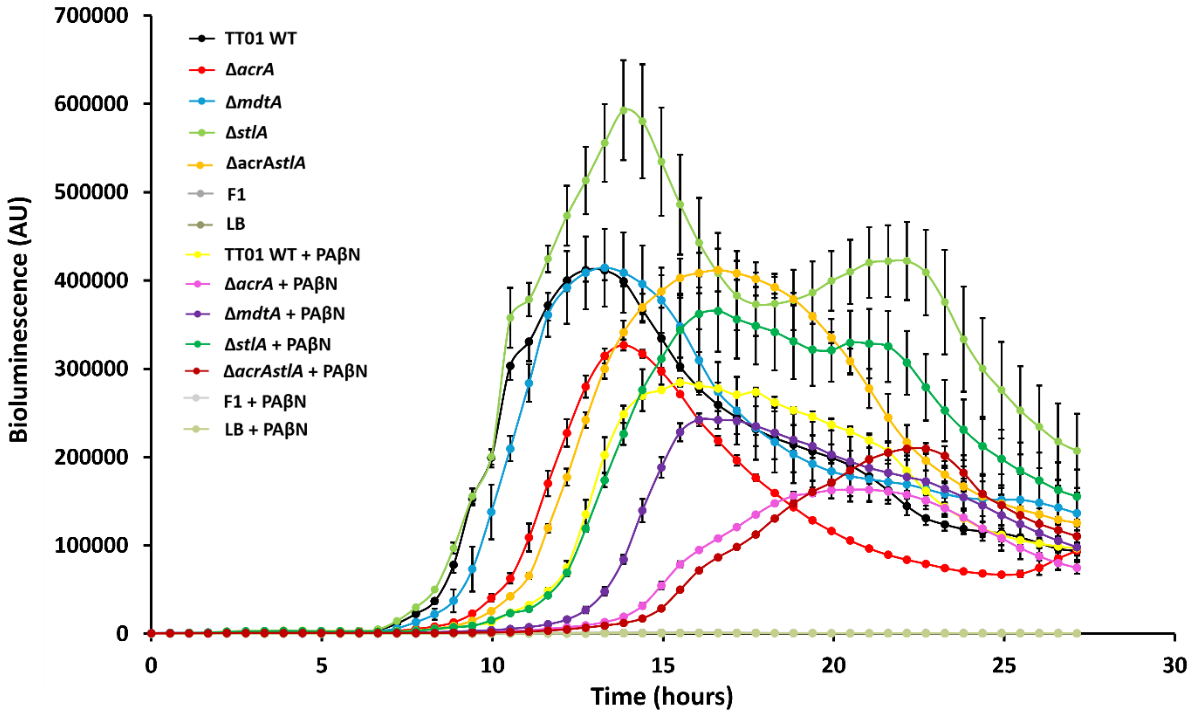

Fig. S5. PAβN decreases bioluminescence across strains, with enhanced suppression in Δ*acrA* and Δ*acrAstlA* mutants. About 10^3 of each *P. laumondii* WT, Δ*acrA*, Δ*mdtA*, Δ*stlA,* and Δ*acrAstlA* strains were inoculated into LB broth in 96-well white microtiter plates, with or without supplementation of 25 $mg.L^{-1}$ PAβN and grown for 27 h at 28°C. *Xenorhabdus nematophila* F1 and uninoculated LB broth were used as negative controls. Bioluminescence was monitored over time using an Infinite M200 microplate reader (Tecan). Data are from three independent biological and experimental replicates and are presented as means ± standard errors of the mean (SEM).

A

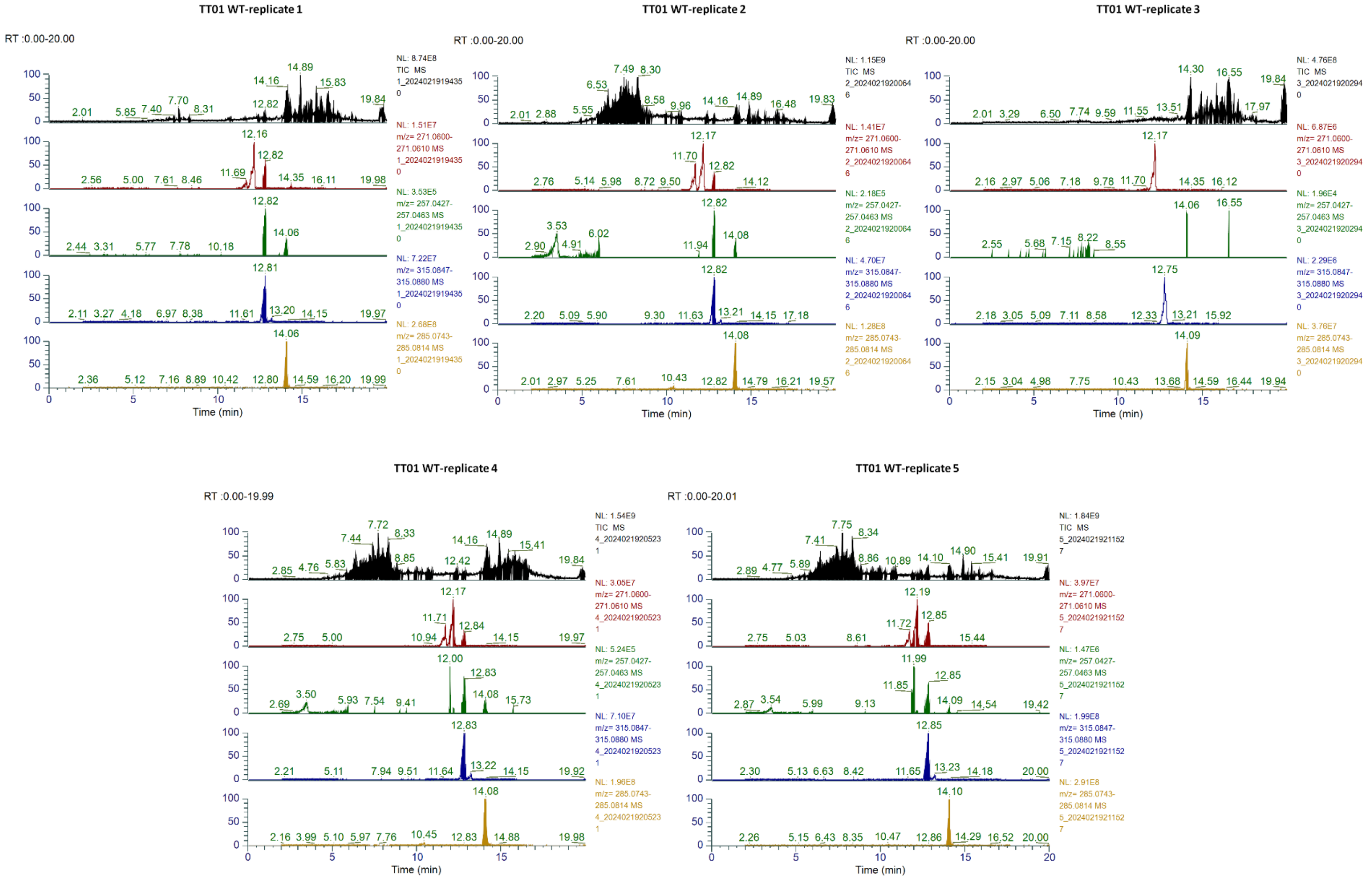
TT01 WT-replicate 1
TT01 WT-replicate 2
TT01 WT-replicate 3
TT01 WT-replicate 4
TT01 WT-replicate 5
RT :0.00-20.00
RT :0.00-19.99
RT :0.00-20.01
Time (min)

B

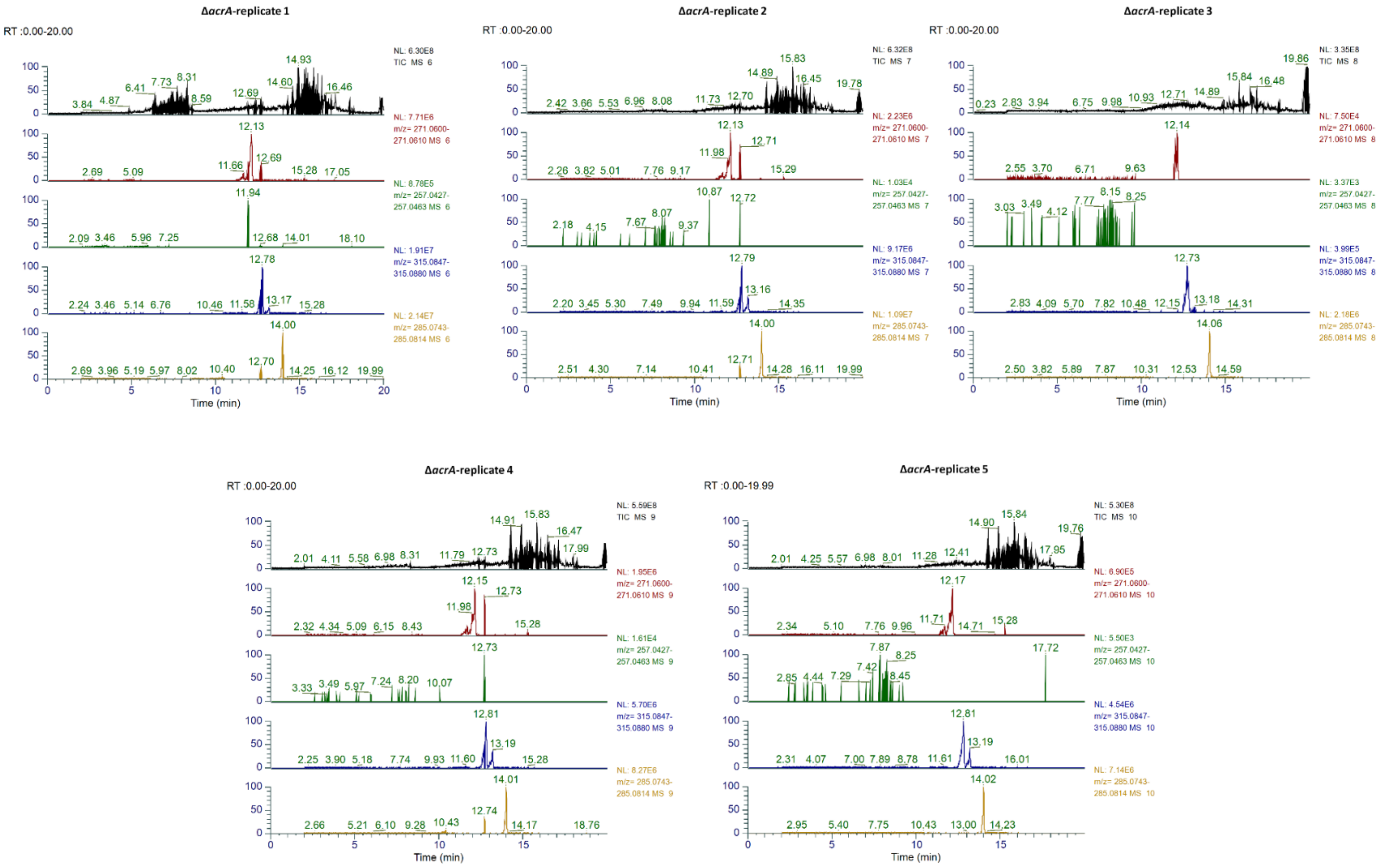

Δ*acrA*-replicate 1
RT :0.00-20.00
Δ*acrA*-replicate 2
RT :0.00-20.00
Δ*acrA*-replicate 3
RT :0.00-20.00
Δ*acrA*-replicate 4
RT :0.00-20.00
Δ*acrA*-replicate 5
RT :0.00-19.99
Time (min)

C

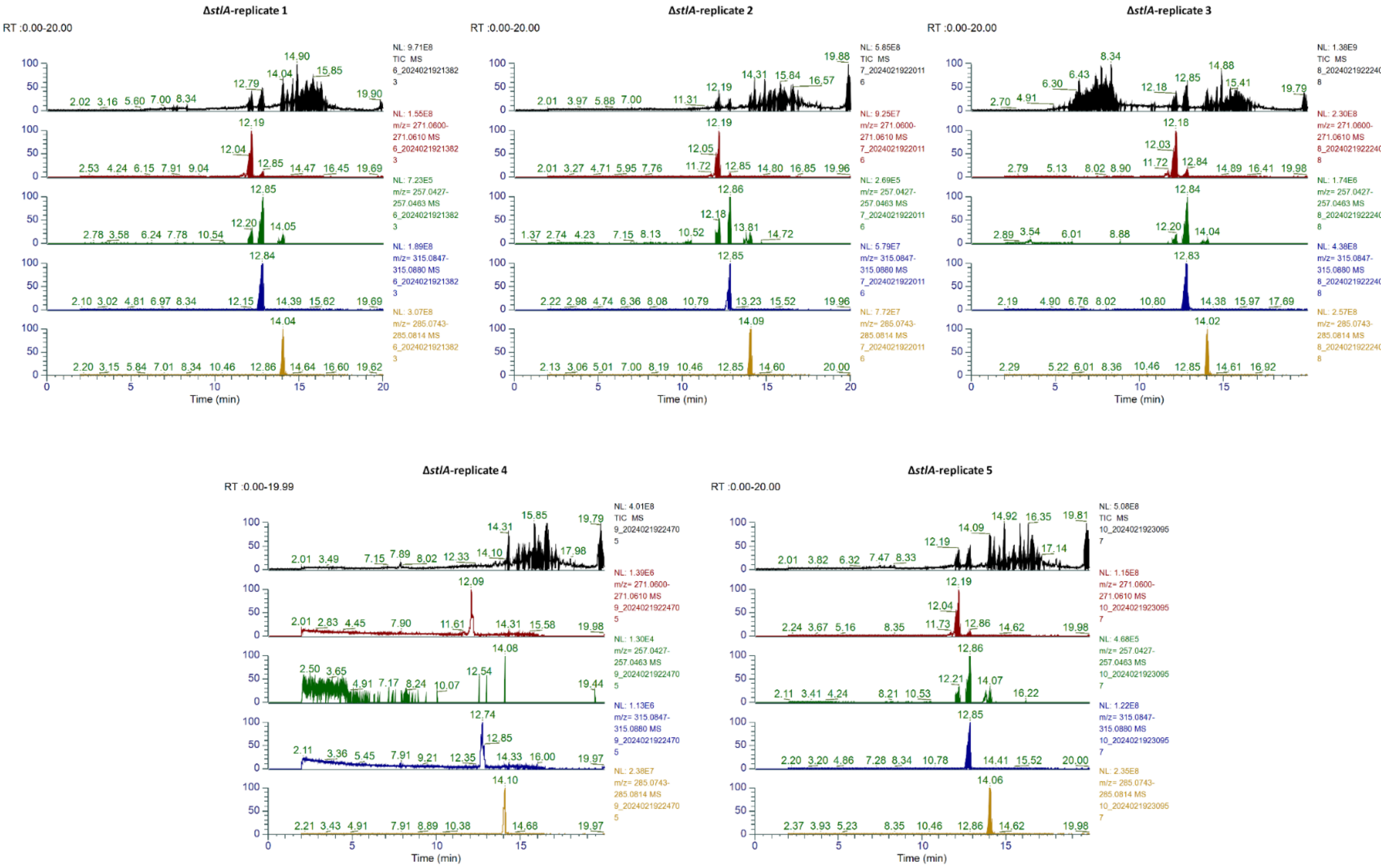
ΔstlA-replicate 1
ΔstlA-replicate 2
ΔstlA-replicate 3
ΔstlA-replicate 4
ΔstlA-replicate 5
RT :0.00-20.00
RT :0.00-19.99
TIC MS
m/z= 271.0600-271.0610 MS
m/z= 257.0427-257.0463 MS
m/z= 315.0847-315.0880 MS
m/z= 285.0743-285.0814 MS
Time (min)

D

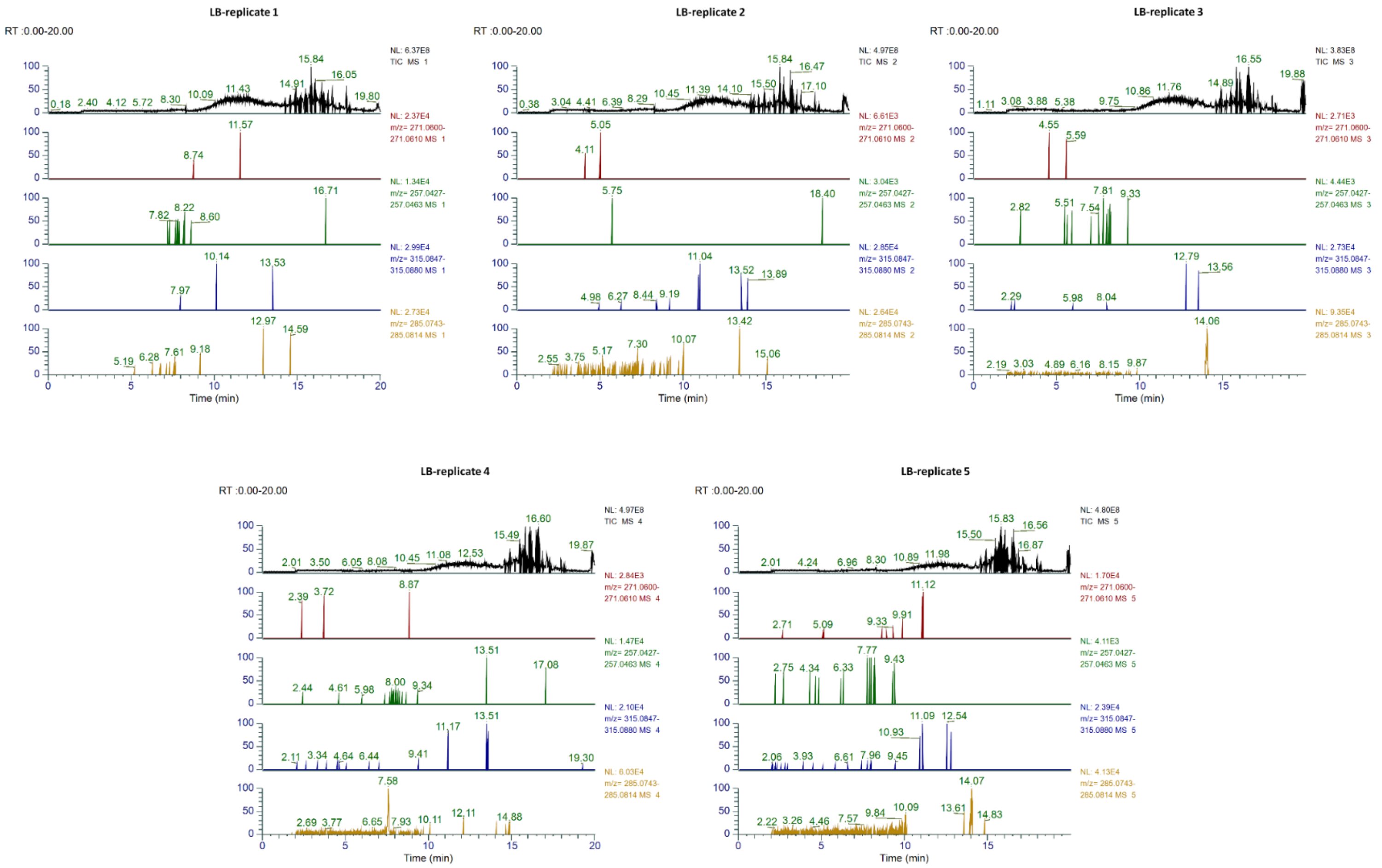
LB-replicate 1
LB-replicate 2
LB-replicate 3
LB-replicate 4
LB-replicate 5
RT :0.00-20.00
TIC MS
m/z= 271.0600-271.0610 MS
m/z= 257.0427-257.0463 MS
m/z= 315.0847-315.0880 MS
m/z= 285.0743-285.0814 MS
Time (min)

Fig. S6. Identification of anthraquinone derivative-mediated pigmentation in extracellular extracts from various *P. laumondii* TT01 strains. HPLC-MS analysis (Total ion chromatograms and extracted ion chromatograms for m/z $[M+H]^+$ = 257.045 Da, 271.060 Da, 285.075 Da, and 315.086 Da, and t_R about 12.82, 12.16, 14.06, and 12.81 min, respectively) were performed on five independent replicates from each of (A) *P. laumondii* WT, (B) Δ*acrA*, (C) Δ*stlA* and (D) LB medium. LB medium was used as a negative control. None of the naturally produced anthraquinone derivatives were detected in the LB medium.

Supplementary Tables

Table S1A. List of 5 genes differentially expressed between *P. laumondii* TT01 WT and Δ*mdtA* grown to exponential (OD_{540} = 0.5-0.7) growth phase in LB medium (Log2 fold change ≥ 1; Adjusted *p*-value (FDR) ≤ 0.05).

Label	Type	Name	Product	Begin	End	Length	Frame	DESid	Normalized average read count	Log2 fold change*	Adjusted *p*-value (FDR)
plu1866	CDS		Similar to nitrilotriacetate monooxygenase component A	2211178	2212509	1332	1	673	437	-1.06	4.05E-02
plu2774	CDS	*mdtA*	MdtABC-TolC efflux pump, multidrug resistance; membrane fusion protein, signal anchor (by similarity)	3287940	3289145	1206	3	673	383	-2.97	1.86E-29
plu2775	CDS	*mdtB*	MdtABC-TolC efflux pump, multidrug resistance; heterodimeric RND-type transporter (by similarity)	3289145	3292291	3147	2	673	671	-4.52	9.63E-41
plu2776	CDS	*mdtC*	MdtABC-TolC efflux pump, multidrug resistance; heterodimeric RND-type transporter (by similarity)	3292288	3295398	3111	1	673	464	-4.13	3.95E-31
plu2777	CDS	*baeS*	Sensor kinase for *mdtABCD* and *acrD* regulation (by similarity)	3295519	3296907	1389	1	673	160	-1.34	4.77E-05

*Ratio Δ*mdtA*/TT01 WT.

Table S1B. List of 12 genes differentially expressed between *P. laumondii* TT01 WT and Δ*mdtA* grown to stationary (OD_{540} = 2.3-4.2) growth phase in LB medium (Log2 fold change ≥ 1; Adjusted *p*-value (FDR) ≤ 0.05).

Label	Type	Name	Product	Begin	End	Length	Frame	DESid	Normalized average read count	Log2 fold change*	Adjusted *p*-value (FDR)
plutRNA0807550D	tRNA		transfert RNA-Asp	807474	807550	77	1	676	134	1.2	3.35E-02
plu1730	CDS		conserved hypothetical protein	2064746	2065195	450	-3	676	343	-1.15	3.35E-02
plu2649	CDS		hypothetical protein	3103565	3103987	423	2	676	3122	-1.11	4.00E-02
plu2686	CDS	*yebY*		3189922	3190275	354	-1	676	115	-1.62	7.67E-03
plu2774	CDS	*mdtA*	MdtABC-TolC efflux pump, multidrug resistance; membrane fusion protein, signal anchor (by similarity)	3287940	3289145	1206	3	676	244	-1.82	2.05E-06
plu2775	CDS	*mdtB*	MdtABC-TolC efflux pump, multidrug resistance; heterodimeric RND-type transporter (by similarity)	3289145	3292291	3147	2	676	406	-2.55	2.53E-19
plu2776	CDS	*mdtC*	MdtABC-TolC efflux pump, multidrug resistance; heterodimeric RND-type transporter (by similarity)	3292288	3295398	3111	1	676	303	-2.16	5.13E-12
plu3489	CDS		hypothetical protein	4085834	4086064	231	2	676	523	1.08	1.73E-03
pluCDS4600716R	CDS		protein of unknown function	4600716	4600796	81	-2	676	224	-1.47	2.90E-02
plu4381	CDS	*dltC*	D-alanine carrier protein DltC	5115942	5116166	225	3	676	142	-1.07	4.20E-02
plutRNA5469493R	tRNA		transfert RNA-Trp	5469493	5469568	76	-1	676	1333	1.47	6.10E-05
plutRNA5469578R	tRNA		transfert RNA-Asp	5469578	5469654	77	-1	676	596	1.35	6.12E-04

*Ratio Δ*mdtA*/TT01 WT.

Table S2A. List of 43 genes differentially expressed between *P. laumondii* TT01 WT and Δ*acrA* grown to exponential (OD_{540} = 0.5-0.7) growth phase in LB medium (Log2 fold change ≥ 1; Adjusted *p*-value (FDR) ≤ 0.05).

Label	Type	Name	Product	Begin	End	Length	Frame	DESid	Normalized average read count	Log2 fold change*	Adjusted *p*-value (FDR)
plu0172	CDS	*yiaO*		182823	183803	981	-2	672	392	-1.45	1.68E-05
plu0173	CDS	*yiaM*		184101	184607	507	3	672	139	-1.19	3.87E-02
plu0225	CDS		Putative TpsA-related protein	230329	235488	5160	-1	672	2128	1.17	1.59E-05
plu0470	CDS	*malP*	maltodextrin phosphorylase	506182	508584	2403	-1	672	347	-1.42	4.35E-05
plu0549	CDS	*cdiB*	CdiB protein (TpsB family)	618577	620268	1692	-1	672	119	-1.77	1.04E-06
plu0769	CDS	*mrfA*	Major fimbrial subunit polypeptide, MrfA	888552	889091	540	3	672	1.29E+04	-3.38	2.16E-17
plu0770	CDS	*mrfB*	Fimbrial pilin protein precursor, MrfB	889175	889723	549	2	672	836	-3.24	3.04E-10
plu0771	CDS	*mrfC*	Outer membrane usher protein MrfC	889743	892298	2556	3	672	1839	-2.96	6.22E-14
plu0772	CDS	*mrfD*	Periplasmic fimbrial chaperone precursor, MrfD	892422	893171	750	3	672	569	-2.45	7.43E-07
plu0773	CDS	*mrfX*	Fimbrial minor pilin protein precursor, MrfX	893196	893789	594	3	672	362	-2.59	5.13E-12
plu0774	CDS	*mrfE*	Minor fimbrial subunit protein, MrfE	893782	894357	576	1	672	225	-2.06	1.50E-06
plu0775	CDS	*mrfF*	Minor fimbrial subunit protein, MrfF	894372	894863	492	3	672	214	-1.34	3.42E-03
plu0776	CDS	*mrfG*	Minor fimbrial subunit protein, MrfG protein	894875	895420	546	2	672	154	-1.4	1.56E-08
plu0970	CDS	*tcdA2*	A component of insecticidal toxin complex, protein TcdA2	1136558	1143892	7335	-3	672	435	1.01	1.57E-04
plu0975	CDS	*hpaB*	4-hydroxyphenylacetate 3-monooxygenase, oxygenase component (4-HPA 3-monooxygenase large component) (4-HPA 3-hydroxylase)	1155152	1156714	1563	-3	672	2624	-1.02	1.16E-04

plu0989	CDS	*hpaG2*	putative C-terminal region of 4-hydroxyphenylacetate degradation bifunctional isomerase/decarboxylase HpaG [Includes: 2-hydroxyhepta-2,4-diene-1,7-dioate isomerase (HHDD isomerase); 5-carboxymethyl-2-oxo-hex-3-ene-1,7-dioate decarboxylase (OPET decarboxylase)]	1172853	1173614	762	-2	672	2585	-1.03	4.04E-02
plu1872	CDS		Putative acetyltransferase	2216252	2216713	462	2	672	170	1.15	7.53E-03
plu1873	CDS		Similar to carbamoylphosphate synthase	2216710	2217873	1164	1	672	336	1.39	4.22E-04
plu1915	CDS	*flgB*	Flagellar basal-body rod protein FlgB (Putative proximal rod protein)	2281971	2282384	414	3	672	108	-2.07	1.64E-07
plu1916	CDS	*flgC*	Flagellar basal-body rod protein FlgC (Putative proximal rod protein)	2282390	2282794	405	2	672	87	-2.26	1.56E-08
plu1917	CDS	*flgD*	Basal-body rod modification protein FlgD (flagellar protein for the initiation of hook assembly)	2282807	2283502	696	2	672	145	-1.95	4.02E-09
plu1918	CDS	*flgE*	Flagellar hook protein FlgE	2283530	2284744	1215	2	672	361	-1.37	3.60E-11
plu1919	CDS	*flgF*	Flagellar basal-body rod protein FlgF (Putative proximal rod protein)	2284763	2285518	756	2	672	289	-1.63	8.74E-08
plu1920	CDS	*flgG*	Flagellar basal-body rod protein FlgG (Distal rod protein)	2285542	2286324	783	1	672	281	-1.08	5.54E-08
plu1939	CDS	*fliO*	flagellar FliO protein	2305963	2306415	453	-1	672	46	-1.48	2.35E-03
plu1940	CDS	*fliN*	Flagellar motor switch protein FliN	2306417	2306836	420	-3	672	82	-1.74	9.21E-06
plu1941	CDS	*fliM*	Flagellar motor switch protein FliM	2306829	2307833	1005	-2	672	213	-2.03	4.38E-07
plu1942	CDS	*fliL*	Flagellar protein FliL	2307839	2308315	477	-3	672	124	-1.62	3.62E-05
plu1943	CDS	*fliK*	Flagellar hook-length control protein FliK	2308489	2309817	1329	-1	672	140	-1.47	1.51E-07
plu1944	CDS	*fliJ*	Flagellar protein FliJ	2309817	2310260	444	-2	672	47	-1.19	8.12E-03
plu1945	CDS	*fliI*	flagellum-specific ATP synthase	2310303	2311667	1365	-2	672	169	-1.45	1.74E-02
plu1946	CDS	*fliH*	Flagellar assembly protein FliH	2311667	2312371	705	-3	672	133	-1.2	1.18E-02

plu1947	CDS	*fliG*	Flagellar motor switch protein FliG	2312364	2313356	993	-2	672	117	-1.43	2.50E-07
plu1948	CDS	*fliF*	flagellar basal-body M-ring protein	2313353	2315056	1704	-3	672	142	-1.43	9.15E-04
plu1954	CDS	*fliC*	Flagellin	2319961	2321028	1068	1	672	1444	-1.17	3.52E-12
plu1969	CDS	*dsdX*	DsdX permease	2341397	2342734	1338	2	672	1108	2.3	1.57E-31
plu1970	CDS	*dsdA*	D-serine dehydratase DsdA	2343419	2344750	1332	2	672	2214	1.65	1.55E-18
plu2453	CDS		Putative TpsA-related protein	2880371	2885434	5064	2	672	187	1.1	7.60E-04
pluCDS3068475D	CDS			3068200	3068475	276	1	672	109	1.1	1.64E-03
plu3741	CDS		putative L-ribulokinase AraB	4412848	4414440	1593	1	672	499	-1.42	5.32E-06
plu3851	CDS	*acrA*	acriflavin resistance protein A precursor	4522214	4523407	1194	2	672	6226	-1.54	2.97E-14
plu3852	CDS	*acrB*	acriflavin resistance protein B	4523435	4526584	3150	2	672	1.05E+04	-6.06	2.65E-138
plu4216	CDS		Conserved hypothetical protein	4931721	4931996	276	-2	672	157	1.1	4.22E-04

*Ratio Δ*acrA*/TT01 WT.

Table S2B. List of 185 genes differentially expressed between *P. laumondii* TT01 WT and Δ*acrA* grown to stationary growth phase (OD_{540} = 2.3-4.2) in LB medium (Log2 fold change ≥ 1; Adjusted *p*-value (FDR) ≤ 0.05).

Label	Type	Name	Product	Begin	End	Length	Frame	DESid	Normalized average read count	Log2 fold change*	Adjusted *p*-value (FDR)
plu0014	CDS	*pts21*	Major phage tail tube protein	14995	15510	516	-1	675	6337	-1.05	5.41E-05
plu0015	CDS	*pts20*	Major phage tail sheath protein	15521	16693	1173	-3	675	1.48E+04	-1.05	6.42E-04
plu0021	CDS	*pts14*	Tail fiber protein of bacteriophage	19522	20649	1128	-1	675	1787	-1	1.56E-05
plu0025	CDS	*pts10*	Phage-related baseplate assembly protein	22597	23439	843	-1	675	446	-1.23	1.99E-04
plu0031	CDS	*pts4*	Tail fiber protein of phage tail-like bacteriocin	26532	27542	1011	3	675	150	-1.73	2.22E-06
plu0033	CDS	*pts2*	Tail fiber assembly protein from bacteriophage	28312	28896	585	-1	675	79	-1.54	8.50E-04
plu0034	CDS	*pts1*	Alternative tail fiber protein of bacteriophage-related plasmid and bacteriophage Product	28944	29999	1056	-2	675	130	-1.94	2.14E-07
plu0082	CDS		conserved hypothetical protein	78376	78669	294	-1	675	417	1	3.34E-05
plu0113	CDS		conserved hypothetical protein	116000	116875	876	-3	675	632	-1.64	3.25E-07
plu0114	CDS		hypothetical protein	117629	118126	498	2	675	409	-1.03	3.67E-04
plu0158	CDS	*amt*	L-arginine:lysine amidinotransferase	163665	164765	1101	3	675	3787	-2.28	2.59E-28
plu0159	CDS		conserved hypothetical protein	164775	165785	1011	3	675	679	-2.24	2.18E-18
plu0160	CDS		conserved hypothetical protein	165859	166740	882	1	675	352	-1.17	1.94E-03
plu0209	CDS	*feoA*	ferrous iron transport protein A	214227	214460	234	-2	675	130	1.32	1.31E-04
plu0318	CDS		Putative AidA protein	341028	341618	591	-2	675	695	-1.12	9.05E-03

plu0362	CDS		Conserved hypothetical protein with DNA binding HTH domain, Fis-type (IPR002197)	388056	388856	801	-2	675	2335	1.05	1.68E-03
plu0370	CDS		Conserved hypothetical protein with basic-leucine zipper domain (IPR004827)	398357	398797	441	-3	675	3474	1.09	1.30E-03
plu0371	CDS		Putative type VI secretion protein, EvpB/VC_A0108, tail sheath (domain IPR010269)	398804	400282	1479	-3	675	1.13E+04	1.19	1.15E-04
plu0462	CDS			493533	495647	2115	3	675	1344	1.16	5.50E-05
plu0463	CDS		conserved hypothetical protein	495650	497008	1359	2	675	2167	1.38	9.56E-05
plu0464	CDS			497025	498170	1146	3	675	1518	1.01	1.61E-04
plu0466	CDS			499391	500536	1146	2	675	1060	1.07	1.07E-04
plu0582	CDS	*bglB*	6-phospho-beta-glucosidase B	660446	661867	1422	-3	675	228	1.02	7.15E-04
plu0734	CDS		conserved hypothetical protein	841205	842320	1116	-3	675	2230	-1.91	5.50E-05
plu0770	CDS	*mrfB*	Fimbrial pilin protein precursor, MrfB	889175	889723	549	2	675	282	-3.1	5.51E-11
plu0771	CDS	*mrfC*	Outer membrane usher protein MrfC	889743	892298	2556	3	675	516	-2.16	4.33E-08
plu0774	CDS	*mrfE*	Minor fimbrial subunit protein, MrfE	893782	894357	576	1	675	158	-1.06	3.57E-02
plu0805	CDS	*tccA3*	Insecticidal toxin complex protein TccA3	930607	933552	2946	1	675	132	1.26	6.33E-03
plu0818	CDS		Some similarities with gp31 protein of Bacteriophage	949226	950371	1146	-3	675	927	1.62	1.46E-07
plu0819	CDS		Some similarities with gp31 protein of Bacteriophage	950408	951553	1146	-3	675	1364	1.73	1.81E-06
plu0820	CDS		Highly similar to unknown protein of Photorhabdus and some similarities with gp30 protein of Bacteriophage	951570	952928	1359	-2	675	1138	1.47	7.94E-06
plu0821	CDS		Truncated gene. Highly similar in N terminal to VgrG and VgrE related protein of *Photorhabdus*	952971	953231	261	-2	675	456	1.62	1.85E-09
plu0822	CDS		Photox, mono-ADP-ribosyltransferase targeting actin; lipid-raft-targeting (LRT) motif	953840	955066	1227	-3	675	1554	1.17	1.25E-06

plu0823	CDS		Truncated gene. Some high similarities with gp31 related protein of *Photorhabdus luminescens*	955147	955380	234	-1	675	393	1.4	6.07E-05
plu0824	CDS		Some similarities with gp31 protein of Bacteriophage	955418	956575	1158	-3	675	2268	1.28	8.32E-05
plu0825	CDS		Putative phage-related protein Gp29 and Gp30	956580	957938	1359	-2	675	3026	1.42	2.47E-05
plu0826	CDS		Some similarities with VgrG and VgrE proteins	957941	960082	2142	-3	675	2870	1.01	3.62E-04
plu0920	CDS			1060242	1060964	723	-2	675	2435	-1.06	7.25E-04
plu0947	CDS		Putative antibiotic biosynthesis monooxygenase	1090244	1090615	372	-3	675	2.39E+04	-1.74	1.65E-10
plu0948	CDS		putative transcriptional regulator	1090996	1091469	474	-1	675	1.64E+04	-1.8	2.22E-06
plu0961	CDS	*tcdB1*	B component of insecticidal toxin complex, protein TcdB1	1103029	1107459	4431	-1	675	3755	1.94	1.14E-07
plu0962	CDS	*tcdA1*	A component of insecticidal toxin complex, protein TcdA1	1107512	1115089	7578	-3	675	3885	1.87	1.59E-11
plu1149	CDS	*cdiA*	CdiA protein (TpsA-related family); presence of the VENN motif and DUF638 domain	1327789	1336602	8814	-1	675	1.88E+04	1.47	3.96E-04
plu1322	CDS		putative 2.4-dihydroxybenzoate monooxygenase	1536760	1538427	1668	1	675	534	-1.31	1.39E-06
plu1367	CDS	*cdiA*	CdiA protein (TpsA-related family); presence of the VENN motif and DUF638 domain	1629806	1638691	8886	-3	675	1.86E+04	1.37	3.58E-04
plu1415	CDS		Protein containing Membrane Attack Complex/Perforin (MACPF) domains	1698682	1700214	1533	1	675	1021	-1.28	3.97E-08
plu1449	CDS	*cydA*	Cytochrome D ubiquinol oxidase subunit I (Cytochrome BD-I oxidase subunit I)	1741045	1742613	1569	1	675	6344	1.68	1.47E-13
plu1450	CDS	*cydB*	Cytochrome D ubiquinol oxidase subunit II (Cytochrome BD-I oxidase subunit II)	1742628	1743767	1140	3	675	5410	1.72	2.10E-16
plu1461	CDS		conserved hypothetical protein	1754451	1754807	357	3	675	389	1.1	3.48E-04
plu1479	CDS	*ybhA*		1772809	1773627	819	-1	675	1912	1.37	5.87E-12
plu1492	CDS			1787965	1790106	2142	1	675	1681	1.01	7.64E-06
plu1493	CDS		conserved hypothetical protein	1790109	1791467	1359	3	675	1805	1.38	2.62E-06

plu1494	CDS			1791482	1792627	1146	2	675	1960	1.21	5.41E-05
plu1517	CDS		Lipase	1817431	1818561	1131	-1	675	1139	-1.24	8.41E-03
plu1563	CDS	*yiaY*		1868761	1869912	1152	1	675	8117	-1.63	1.49E-19
plu1576	CDS	*cipA*	Crystalline inclusion protein CipA	1883974	1884288	315	-1	675	3.75E+04	-1.5	9.78E-04
plu1577	CDS		Hypothetical protein	1885002	1885823	822	3	675	939	-1.1	2.97E-03
plu1627	CDS		putative integral membrane protein	1942624	1943259	636	-1	675	382	-1.14	1.58E-02
plu1628	CDS		conserved hypothetical protein	1943264	1944076	813	-3	675	3830	-1.34	3.02E-06
plu1712	CDS			2042171	2043193	1023	-3	675	152	-1.2	3.79E-03
plu1713	CDS			2043313	2044155	843	-1	675	103	-1.92	3.27E-04
plu1715	CDS		conserved hypothetical protein	2045078	2045980	903	-3	675	117	-1.12	4.22E-03
plu1718	CDS		conserved hypothetical protein	2049818	2051122	1305	-3	675	407	-1.49	8.80E-06
plu1728	CDS		conserved hypothetical protein	2062264	2063655	1392	-1	675	2182	-1.05	2.09E-04
plu1730	CDS		conserved hypothetical protein	2064746	2065195	450	-3	675	391	-1.69	1.77E-03
plu1840	CDS		Conserved hypothetical protein	2187702	2188778	1077	3	675	1249	-1.64	2.96E-03
plu1892	CDS		Similar to C-terminal region of killer protein of pyocin S3	2253579	2254067	489	-2	675	934	1.11	1.70E-06
plu1959	CDS	*xylB*	xylulose kinase	2328669	2330180	1512	-2	675	3005	-1.1	2.94E-04
plu1960	CDS		putative xylitol (sorbitol) dehydrogenase	2330184	2331212	1029	-2	675	5510	-1.13	4.53E-04
plu2001	CDS		putative transcription regulator, LuxR family	2372413	2373108	696	-1	675	282	1.75	1.55E-05
plu2002	CDS		putative transcription regulator, LuxR family	2373256	2373927	672	-1	675	356	1.59	2.89E-08

plu2003	CDS		putative transcriptional regulator, LuxR family	2373955	2374635	681	-1	675	398	1.41	4.61E-06
plu2004	CDS		putative transcriptional regulator, LuxR family	2374927	2375607	681	-1	675	5504	1.08	1.65E-09
plu2005	CDS		putative transcriptional regulator, LuxR family	2375637	2376317	681	-2	675	4215	1.16	1.43E-09
plu2006	CDS		putative transcriptional regulator, LuxR family	2376348	2377028	681	-2	675	3009	1.16	1.80E-07
plu2047	CDS	*ISPlu3U*	Transposase, IS630 family	2418061	2419086	1026	-1	675	64	-1.57	1.19E-02
plu2048	CDS		conserved hypothetical protein	2419182	2419988	807	-2	675	2511	-1.12	5.99E-04
plu2049	CDS		conserved hypothetical protein	2420828	2422039	1212	2	675	1082	-1.25	3.03E-05
plu2075	CDS		putative 3-oxoacyl-[acyl-carrier protein] reductase	2458486	2459178	693	1	675	147	-1.35	1.20E-02
plu2125	CDS		hypothetical protein	2510368	2510604	237	-1	675	2390	-1.15	3.96E-04
plu2163	CDS	*stlC*	Cyclase StlC involved in stilben biosynthesis	2548765	2549712	948	1	675	1.91E+04	-1.77	3.67E-12
plu2164	CDS	*stlD*	Cinnamoyl-CoA condensing ketosynthase StlD involved in stilben biosynthesis	2549713	2550861	1149	1	675	3.94E+04	-1.57	5.46E-21
plu2165	CDS	*stlE*	Acyl carrier protein (ACP) StlE involved in stilben biosynthesis	2550941	2551183	243	2	675	6415	-1.61	1.80E-22
plu2217	CDS		putative 3-oxoacyl-[acyl-carrier-protein] synthase	2605416	2606681	1266	3	675	318	-1.95	1.24E-15
plu2218	CDS		conserved hypothetical protein	2606687	2607463	777	2	675	82	-2.53	5.08E-04
plu2220	CDS		conserved hypothetical protein	2607774	2608334	561	3	675	297	-2.23	1.03E-03
plu2221	CDS		conserved hypothetical protein	2608346	2609368	1023	2	675	990	-2.16	8.47E-08
plu2222	CDS		Putative nematicidal protein 2. Probable membrane protein	2609512	2614389	4878	1	675	631	-1.47	2.93E-14
plu2234	CDS	*stlA*	Phenylalanine amonia-lyase StlA involved in stilben biosynthesis	2626090	2627688	1599	-1	675	2303	-2.3	2.75E-25
plu2236	CDS			2630027	2631262	1236	-3	675	960	-1.78	8.87E-12

plu2237	CDS		conserved hypothetical protein	2631904	2632851	948	1	675	125	-1.99	2.46E-03
plu2256	CDS		conserved hypothetical protein	2649009	2649305	297	3	675	1374	-2.79	3.61E-06
plu2303	CDS		putative tail fiber protein	2704727	2705602	876	-3	675	4432	-1.35	4.40E-15
plu2355	CDS		hypothetical protein	2774021	2774488	468	-3	675	303	-1.45	2.41E-06
plu2568	CDS		Similar to unknown Hcp-like protein of *Escherichia coli*	3011929	3012408	480	-1	675	388	1.71	1.31E-05
plu2657	CDS	*pbgP4*	Conserved hypothetical protein PbgP4	3110057	3110950	894	-3	675	249	1.06	3.85E-03
plu2727	CDS	*entA*	Enterobactin synthetase component A	3234194	3234940	747	-3	675	433	-1.37	1.14E-08
plu2728	CDS	*entB*	Isochorismatase (2,3-dihydro-2,3-dihydroxybenzoate synthetase)	3235008	3235661	654	-2	675	159	-1.04	3.70E-02
plu2729	CDS	*entE*	2,3-dihydroxybenzoate--[carrier protein] ligase (enterobactin synthetase component E)	3235684	3237303	1620	-1	675	821	-1.3	5.83E-05
plu3008	CDS		putative phage tail fiber assembly protein	3508362	3509114	753	3	675	616	-2.07	4.72E-10
plu3009	CDS		putative phage tail fiber assembly protein	3509124	3509636	513	3	675	209	-1.75	3.93E-04
plu3012	CDS		putative tail fiber assembly protein from bacteriophage	3512834	3513403	570	-3	675	835	-1.88	5.80E-06
plu3013	CDS		putative tail fiber assembly protein from bacteriophage	3513460	3514161	702	-1	675	648	-1.95	2.61E-10
plu3022	CDS			3523655	3524143	489	2	675	199	-1.13	3.31E-02
plu3024	CDS		putative tail fiber protein from a lambdoid prophage	3524613	3525239	627	-2	675	1884	-2.21	5.63E-12
plu3025	CDS		putative Sc/SvQ protein of Escherichia coli plasmid p15B	3525239	3526675	1437	-3	675	2646	-2.37	2.06E-11
plu3026	CDS		Putative bacteriophage protein	3526678	3527250	573	-1	675	720	-2.25	6.82E-06
plu3027	CDS		putative bacteriophage protein	3527247	3528440	1194	-2	675	2365	-1.81	1.46E-07
plu3028	CDS			3528433	3528780	348	-1	675	654	-2.02	1.29E-07

plu3029	CDS			3528777	3529532	756	-2	675	1734	-1.76	2.49E-06
plu3030	CDS		Putative bacteriophage protein	3529529	3530485	957	-3	675	1362	-2.07	1.89E-14
plu3031	CDS		Putative bacteriophage protein	3530576	3530881	306	-3	675	269	-1.92	2.14E-05
plu3032	CDS		conserved hypothetical protein	3530866	3531471	606	-1	675	642	-1.82	1.93E-07
plu3033	CDS			3531474	3533243	1770	-2	675	2292	-1.95	1.86E-09
pluCDS3533233R	CDS		conserved protein of unknown function	3533233	3533382	150	-1	675	355	-2.13	5.41E-05
plu3034	CDS		Putative bacteriophage protein	3533436	3533846	411	-2	675	1206	-2.1	3.01E-06
plu3035	CDS		Putative bacteriophage protein	3533960	3534400	441	-3	675	4611	-2.09	1.16E-11
plu3036	CDS		Putative bacteriophage protein	3534410	3535879	1470	-3	675	1.38E+04	-2.02	8.87E-10
plu3037	CDS			3535883	3536446	564	-3	675	1849	-2.17	1.86E-09
plu3043	CDS	*bacA*	BacA, prephenate decarboxylase involved in 2,5-dihydrophenylalanine, precursor of 2,5-dihydroxystilbene	3542532	3543110	579	3	675	1514	1	1.12E-03
plu3064	CDS		Putative TpsA-related protein	3566742	3571829	5088	-2	675	1772	1.36	5.31E-09
plu3097	CDS		conserved hypothetical protein	3614906	3615562	657	-3	675	127	-1.58	2.62E-02
plu3102	CDS		putative methyltransferase	3621255	3622079	825	-2	675	1597	1.51	3.12E-05
plu3103	CDS		putative cysteine desulfurase (NifS protein homolog)	3622091	3623257	1167	-3	675	1212	1.53	6.08E-08
plu3104	CDS		conserved hypothetical protein	3623257	3624141	885	-1	675	1104	1.58	1.00E-09
plu3129	CDS		conserved hypothetical protein	3677817	3678092	276	3	675	515	-1.8	1.64E-06
plu3130	CDS		Probable non-ribosomal peptide synthetase	3678528	3688463	9936	-2	675	1.12E+04	-1.69	6.31E-05

plu3134	CDS		putative 4-hydroxybenzoate transporter	3692924	3694246	1323	-3	675	303	-1.71	1.45E-07
plu3260	CDS		putative Type VI secretion protein, VC_A0114 (IPR010263)	3860551	3861900	1350	-1	675	424	1.2	8.10E-03
plu3261	CDS		putative type VI secretion protein, EvpB/VC_A0108, tail sheath (domain IPR010269)	3861916	3863442	1527	-1	675	523	1.74	1.51E-06
plu3262	CDS		putative Type VI secretion system, VipA, VC_A0107 or Hcp2 (domain IPR008312)	3863474	3863971	498	-3	675	199	1.6	2.24E-06
plu3558	CDS		conserved hypothetical protein	4191700	4193004	1305	-1	675	1874	-1.08	2.62E-06
plu3569	CDS		TpsB protein	4208442	4210121	1680	-2	675	86	1.31	2.51E-03
plu3593	CDS		conserved hypothetical protein	4235193	4236086	894	-2	675	114	1.79	8.54E-04
plu3594	CDS		Putative TpsA-related protein	4236073	4242480	6408	-1	675	2786	1.44	2.97E-17
plu3595	CDS			4242432	4242791	360	-2	675	128	2.02	1.40E-04
plu3613	CDS			4261382	4262680	1299	2	675	188	-2.7	1.86E-09
plu3718	CDS	*cdiA*	CdiA protein (TpsA-related family); presence of the VENN motif and DUF638 domain	4374057	4383140	9084	-2	675	8434	1.37	2.03E-03
plu3794	CDS		conserved hypothetical protein (some similarities with Hemolysin of Fusobacterium nucleatum subsp. nucleatum)	4458146	4459174	1029	2	675	290	-1.29	1.10E-02
plu3795	CDS		conserved hypothetical protein	4459644	4460093	450	-2	675	2.32E+04	-2.02	4.18E-14
plu3832	CDS		putative transmembrane protein	4501753	4502364	612	-1	675	79	-1.45	4.59E-03
plu3851	CDS	*acrA*	acriflavin resistance protein A precursor	4522214	4523407	1194	2	675	4042	-1.19	8.48E-08
plu3852	CDS	*acrB*	acriflavin resistance protein B	4523435	4526584	3150	2	675	7364	-4.51	1.46E-73
plu3922	CDS		hypothetical protein	4600912	4601094	183	-1	675	247	-1.4	1.25E-02
plu3934	CDS		conserved hypothetical protein	4616440	4618194	1755	-1	675	268	-1.34	2.72E-04

plu3935	CDS		conserved hypothetical protein	4618718	4620466	1749	-3	675	118	-1.26	1.21E-02
plu3994	CDS	*yqjD*	Putative membrane protein YqjD of *Escherichia coli*, paralogous to elaB and ygaM	4682528	4682833	306	2	675	1.06E+05	-1.02	2.89E-03
plu3995	CDS	*yqjE*		4682835	4683236	402	3	675	3.42E+04	-1.12	1.28E-08
plu3996	CDS	*yqjK*		4683229	4683489	261	1	675	2847	-1.47	1.60E-06
plu4185	CDS		Putative regulator of antibiotic production	4897234	4897935	702	-1	675	83	-1.16	2.64E-02
plu4186	CDS	*antI*	Hydrolase/peptidase AntI involved in anthraquinone biosynthesis (type II polyketide synthase)	4898888	4900042	1155	-3	675	1549	-1.41	1.72E-04
plu4187	CDS	*antH*	Cyclase/aromatase AntH involved in anthraquinone biosynthesis (type II polyketide synthase)	4900062	4901504	1443	-2	675	3009	-1.42	5.99E-04
plu4188	CDS	*antG*	CoA ligase AntG involved in anthraquinone biosynthesis (type II polyketide synthase)	4901504	4903051	1548	-3	675	1706	-1.08	9.65E-05
plu4189	CDS	*antF*	Acyl carrier protein AntF involved in anthraquinone biosynthesis (type II polyketide synthase)	4903075	4903323	249	-1	675	1096	-1.24	6.87E-05
plu4190	CDS	*antE*	Ketosynthase KS-beta AntE involved in anthraquinone biosynthesis (type II polyketide synthase)	4903358	4904473	1116	-3	675	2258	-1.19	3.39E-11
plu4191	CDS	*antD*	Ketosynthase KS-alpha AntD involved in anthraquinone biosynthesis (type II polyketide synthase)	4904466	4905752	1287	-2	675	2350	-1.16	6.22E-14
plu4192	CDS	*ant C*	Cyclase AntC involved in anthraquinone biosynthesis (type II polyketide synthase)	4906404	4907219	816	-2	675	730	-1.49	1.05E-02
plu4193	CDS	*antB*	Phosphopantetheinyl transferase AntB involved in anthraquinone biosynthesis (type II polyketide synthase)	4907212	4907925	714	-1	675	1194	-1.94	4.47E-08
plu4194	CDS	*antA*	Ketoreductase AntA involved in anthraquinone biosynthesis (type II polyketide synthase)	4907928	4908704	777	-2	675	4166	-1.54	3.51E-26
plu4211	CDS		Putative Type VI secretion system effector, Hcp (IPR008514)	4925955	4926446	492	-2	675	2.31E+04	1.39	6.79E-12
plu4230	CDS		conserved hypothetical protein	4952453	4953391	939	2	675	164	-1.04	3.68E-02
plu4238	CDS		conserved hypothetical protein	4960560	4961522	963	3	675	345	-1.77	1.45E-05
plu4261	CDS		conserved hypothetical protein	4985678	4986637	960	2	675	330	2.61	4.54E-19

plu4262	CDS		putative L-ornithine 5-monooxygenase (L-ornithine N5-oxygenase)	4986690	4988015	1326	3	675	69	2.74	4.87E-06
plu4265	CDS		putative methionyl-tRNA synthetase	4989752	4991245	1494	2	675	59	2.57	1.81E-06
plu4269	CDS		putative amino acid decarboxylase	4994680	4996128	1449	1	675	94	2.48	1.33E-05
plu4274	CDS		putative monoamine regulon transcriptional regulator MoaR	5000547	5001206	660	3	675	128	1.7	9.88E-06
plu4351	CDS	*yeiN*	Hypothetical protein yeiN	5084097	5085035	939	-2	675	1815	1.05	3.78E-05
plu4352	CDS	*yeiC*	Putative kinase YeiC protein of *Escherichia coli*	5085038	5086123	1086	-3	675	946	1.32	1.09E-11
plu4393	CDS		conserved hypothetical protein	5131421	5131987	567	-3	675	1522	-1.96	4.37E-11
plu4488	CDS	*tccC7*	Insecticidal toxin complex protein TccC7	5242615	5245425	2811	1	675	565	1.08	4.62E-04
plu4565	CDS	*pcfD*	Carbamoyl transferase, NodU family, involved in the induction of *Photorhabdus* cell clumping	5329532	5331229	1698	-3	675	2186	-1.94	4.33E-08
plu4567	CDS	*pcfB*	Argininosuccinate synthase, involved in the induction of *Photorhabdus* cell clumping	5332053	5333243	1191	-2	675	210	-3.03	1.04E-17
plu4568	CDS	*pcfA*	cysteine synthase, O-acetylserine sulfhydrolase subunit, involved in the induction of *Photorhabdus* cell clumping	5333233	5334261	1029	-1	675	250	-2.85	1.00E-12
plu4600	CDS		Conserved hypothetical protein	5366825	5368558	1734	2	675	2970	1.44	3.15E-08
plu4601	CDS			5368573	5370549	1977	1	675	3397	1.44	3.98E-07
plu4602	CDS		Conserved hypothetical protein	5370546	5371037	492	3	675	652	1.2	5.70E-07
plu4603	CDS		conserved hypothetical protein	5371040	5371384	345	2	675	233	1.2	3.54E-05
plu4604	CDS		conserved hypothetical protein	5371386	5371994	609	3	675	468	1.46	6.59E-07
plu4607	CDS		conserved hypothetical protein	5373170	5373802	633	2	675	145	1.21	2.30E-03
plu4827	CDS			5602692	5603162	471	3	675	242	1.23	1.75E-04

plu4831	CDS	*galE*	UDP-glucose 4-epimerase (Galactowaldenase) (UDP-galactose 4-epimerase)	5605994	5607016	1023	2	675	136	1.08	1.77E-03
plu4892	CDS			5665879	5666832	954	-1	675	1215	-2.05	3.15E-08
plu4894	CDS			5668798	5669751	954	-1	675	188	-1.21	7.28E-03
plu4895	CDS			5670464	5671417	954	-3	675	420	-1.29	3.15E-03

*Ratio Δ*acrA*/TT01 WT.

Colors highlight some functional categories:

Secondary metabolite biosynthesis: Stilbenes, fatty acids, anthraquinones and enterobactin.

Quorum sensing: Putative LuxR-type regulators, cellular clumping and fimbrial pilin formation.

Toxic/virulent system: Type VI secretion system, insecticidal toxin and two partner system (Tps).

Table S3. Strains and plasmids used in this study.

Strain or plasmid	Genotype and relevant characteristics	Reference
Strains		
Photorhabdus laumondii		
TT01	Wild-type isolated from *Heterorhabditis bacteriophora* nematode TH01	(4)
Δ*acrA*	TT01 *acrA*::Km	(5)
Δ*mdtA*	TT01 *mdtA*::ΩCm	(6)
Δ*acrA*-like	TT01 *acrA*-like::ΩCm	(5)
Δ*stlA*	TT01 *stlA*::ΩCm	(7)
Δ*acrAstlA*	TT01 *acrA*::Km *stlA*::ΩCm	This study
Xenorhabdus nematophila		
F1	Wild-type isolated from *Steinernema carpocapsae* nematode Plougastel (Brittany)	Laboratory collection
Escherichia coli		
XL1-Blue MRF’	Δ(*mcrA*)*183* Δ(*mcrCB-hsdSMR-mrr*)*173 endA1 supE44 thi-1 recA1 gyrA96 relA1 lac* [*F´ proAB lacIqZ*Δ*M15* Tn*10* (Tet^r)]	Stratagene
WM3064	*thrB*1004 *pro thi rpsl hsdS lac*ZΔM15 RP4-1360Δ(*araBAD*)567 Δ*dapA1341*::[erm pir (wt)] donor strain	(8)
Plasmids		
pBBR1-MCS5	Gm^r *mob* broad-host range vector	(9)
pBBR1-*acrAB*	pBBR1-MCS5 carrying *acrAB* operon from TT01 WT under the control of lactose promoter P_{lac} constitutively expressing *acrAB*	(5)
pBBR1-*stlA*	pBBR1-MCS5 carrying *stlA* gene from TT01 WT under the control of lactose promoter P_{lac} constitutively expressing *stlA*	(7)

Table S4. Primers used for the RT-qPCR analysis.

Category	Regions or genes	Primers	Sequences 5’-3’
**Reference**	*recA*	F-recA-TT01q	GTTCAATGGACGTTGAAACTATCTC
		R-recA-TT01q	ATCAACACCCAACTTCTTAGCATAG
**Internal control**	*gyrB*	F-gyrB-TT01q	ATACACGAAGAAGAAGGTGTTTCAG
		R-gyrB-TT01q	TACCTGTCTGTTCAGTTTCTCCAAC
**Anthraquinone biosynthesis**	*antA*	F-antA-TT01q	CTTATTCTGCTTCTAAGCATGGTGT
	(*plu4194*)	R-antA-TT01q	TCTTCTACTCTTCTTTTCGCTTCTG
	O-methyltransferase	F-plu4895-TT01q	ACTTGCTGATCCTAATAGCGTAGAA
	(*plu4895*)	R-plu4895-TT01q	CCCTTATTACTATCCAAATGCTCAA
**Stilbene biosynthesis**	*stlA*	F-stlA-TT01q	AAATATTGGAACGGGAAGTTATCTC
	(*plu2234*)	R-stlA-TT01q	GAGAGAAACGGTTATCCATCATAAG
	stlC	F-stlC-TT01q	TAAGAGTGAAGAAGGAAAGCGTTTA
	(*plu2163*)	R-stlC-TT01q	TCTTTTTCCTGTCATTAGCCATATC
	ST epoxide	F-plu2236-TT01q	AATCCCGGTTTTATAATGAACGTAT
	(*plu2236*)	R-plu2236-TT01q	ATATGTTCATAACGGACGAATGTTT
	bacA	F-bacA-TT01q	CCCGATAATCTAAGAATTGCAGATA
	(*plu3043*)	R-bacA-TT01q	ATTTGAATTGGTCGAATATGAGAAA
**Fatty acid biosynthesis**	Acyl-carrier	F-plu2217-TT01q	GAGTAATCTTATCACCATGCCAAGT
	(*plu2217*)	R-plu2217-TT01q	ATGCATTCAGTACATCAATACTCCA

**Enterobactin biosynthesis**	*entE*	F-entE-TT01q	CTTGATCAACAATCATCTGCATTAG
	(*plu2729*)	R-entE-TT01q	GACGAAAGTACAATCAGACTTGGAT
	Putative 4-hydroxybenzoate transporter	F-plu3134-TT01q	GTATTAATGGGGTATATTGCACCAG
	(*plu3134*)	R-plu3134-TT01q	CAGATATTGTCAACACTGACTTTCG
**Quorum sensing**	Putative *luxR*-type	F-plu2001-TT01q	TCATTCAGTTACCGGAAAACTTTAG
	(*plu2001*)	R-plu2001-TT01q	ACACAGTGATTATCCTCACTCATCA
	pcfA	F-pcfA-TT01q	ATCGATATTACCTTGAAAGTTGCAG
	(*plu4568*)	R-pcfA-TT01q	TGTGTCGATATAACGTTCTCCACTA
**Type VI secretion system**	*tssB*	F-plu3262-TT01q	AATTATTGGTCATGGGGGATTATAG
	(*plu3262*)	R-plu3262-TT01q	ATTTGTTCAGGCTCGAAGTCTTTTA
	VIP2-ART	F-plu0822-TT01q	ACGTTAAGCATCAAGCTATTGAAAC
	(*plu0822*)	R-plu0822-TT01q	GCAATGATGTTTCCCTCTGTAGTAT
**Two partner system**	*tpsA*	F-tpsA-TT01q	CCCCTATGAATATGAAGATGAACAC
	(*plu3594*)	R-tpsA-TT01q	TCAGTATCAATAATGTCACCACCAG
	tpsB	F-tpsB-TT01q	CTAATGTAACGATCCCTTATGGCTA
	(*plu3569*)	R-tpsB-TT01q	CTGAACTGTAGTTAATGCCAAGGTT
**Fimbrial pilin formation**	*mrfB*	F-mrfB-TT01q	TCCGTTTGATTAACTGTGTACTGAC
	(*plu0770*)	R-mrfB-TT01q	TGCCGTACATGTCTGTAATTTGTAA
**Flagellar formation**	*fliC*	F-fliC-TT01q	CTTGAATAGATCCCAGGGTACTTTG
	(*plu1954*)	R-fliC-TT01q	TAGAGATACCGTCATTAGCGTTACG

	fliF (*plu1948*)	F-fliF-TT01q	ATGAGTGCCGCAACAACC
		R-fliF-TT01q	GATAATCGGGGCTGCGTAAC
**Bacteriophage formation**	*pts1* (*plu0034*)	F-pts1-TT01q	AGCAATTCATATAGCTCATCCTGAC
		R-pts1-TT01q	TGGAATACCAACAAGAATATCATCC
	Putative phage tail fiber assembly (*plu3008*)	F-plu3008-TT01q	CTGACAAAGCCGATTTAAATGATTA
		R-plu3008-TT01q	CCCGTGTATATATCCGACTGCTATT
	Putative bacteriophage (*plu3036*)	F-plu3036-TT01q	TGAGTAAAGAAGCCAATCTTAATGG
		R-plu3036-TT01q	GTTTCTGCTTTGGTAGATTCGATAG
**Monooxygenase**	*sidA* (*plu4262*)	F-sidA-TT01q	ATAAAGTCGTTCGCTATTCAACATC
		R-sidA-TT01q	TGTCGCAGAGATAAAGGAATATAGG
**Permease**	*dsdX* (*plu1969*)	F-dsdX-TT01q	TTTAAAAGTCAGTGGCCTTAGTGAT
		R-dsdX-TT01q	TATCTGGATATAACGGCAACATAGG
**Unknown function**	*yebY* (*plu2686*)	F-yebY-TT01q	GGTGAGTAAGCTACAATTTGGTGAT
		R-yebY-TT01q	ACTTAGCATTCACTTTCCCTACCTT
**AcrAB efflux pump**	*acrA* (*plu3851*)	F-acrA-TT01q	TTATGTGGATGTCACTCAGTCAAGT
		R-acrA-TT01q	AATATTGCACGGATAGTGATAGAGC
	acrB (*plu3852*)	F-acrB-TT01q	TATCTGGTTAAATCCTGACAAGCTC
		R-acrB-TT01q	GAATATTACTGAACTCTTCCGGTGA

References

1. Yan, L., 2024. _ggvenn: Draw Venn Diagram by 'ggplot2'_. R package version 0.1.16, commit 0d6914a6134b2cae60051cbc1dc9850c2a2077e4, <https://github.com/yanlinlin82/ggvenn>.
2. R Core Team (2021). R: A language and environment for statistical computing. R Foundation for Statistical Computing, Vienna, Austria. URL https://www.R-project.org/.
3. Mouammine A, Pages S, Lanois A, Gaudriault S, Jubelin G, Bonabaud M, Cruveiller S, Dubois E, Roche D, Legrand L, Brillard J, Givaudan A. 2017. An antimicrobial peptide-resistant minor subpopulation of *Photorhabdus luminescens* is responsible for virulence. Sci Rep 7:43670.
4. Duchaud E, Rusniok C, Frangeul L, Buchrieser C, Givaudan A, Taourit S, Bocs S, Boursaux-Eude C, Chandler M, Charles JF, Dassa E, Derose R, Derzelle S, Freyssinet G, Gaudriault S, Medigue C, Lanois A, Powell K, Siguier P, Vincent R, Wingate V, Zouine M, Glaser P, Boemare N, Danchin A, Kunst F. 2003. The genome sequence of the entomopathogenic bacterium *Photorhabdus luminescens*. Nat Biotechnol 21:1307-13.
5. Hadchity L, Lanois A, Kiwan P, Nassar F, Givaudan A, Abi Khattar Z. 2021. AcrAB, the major RND-type efflux pump of *Photorhabdus laumondii*, confers intrinsic multidrug-resistance and contributes to virulence in insects. Environ Microbiol Rep 13:637-648.
6. Abi Khattar Z, Lanois A, Hadchity L, Gaudriault S, Givaudan A. 2019. Spatiotemporal expression of the putative MdtABC efflux pump of *Phtotorhabdus luminescens* occurs in a protease-dependent manner during insect infection. PLoS One 14:e0212077.
7. Hadchity L, Houard J, Lanois A, Payelleville A, Nassar F, Gualtieri M, Givaudan A, Abi Khattar Z. 2023. The AcrAB efflux pump confers self-resistance to stilbenes in *Photorhabdus laumondii*. Res Microbiol 174:104081.
8. Paulick A, Koerdt A, Lassak J, Huntley S, Wilms I, Narberhaus F, Thormann KM. 2009. Two different stator systems drive a single polar flagellum in *Shewanella oneidensis* MR-1. Mol Microbiol 71:836-50.
9. Kovach ME, Elzer PH, Hill DS, Robertson GT, Farris MA, Roop RM, 2nd, Peterson KM. 1995. Four new derivatives of the broad-host-range cloning vector pBBR1MCS, carrying different antibiotic-resistance cassettes. Gene 166:175-6.
